# Efficacy of an upper room ultraviolet-C (UV-C) technology versus far UV-C light technologies in reducing aerosolized bacteriophage MS2

**DOI:** 10.1017/ash.2025.10107

**Published:** 2025-09-15

**Authors:** Samir Memic, Jennifer L. Cadnum, Curtis J. Donskey

**Affiliations:** 1 Department of Systems Biology, Case Western Reserve University School of Medicine, Cleveland, OH, USA; 2 Research Service, Louis Stokes Cleveland VA Medical Center, Cleveland, OH, USA; 3 Geriatric Research, Education and Clinical Center, Louis Stokes Cleveland VA Medical Center, Cleveland, OH, USA; 4 Department of Medicine, Case Western Reserve University School of Medicine, Cleveland, OH, USA

## Abstract

In a room with 6 air changes per hour, an upper room ultraviolet-C (UV-C) and 2 far UV-C technologies were similarly effective in reducing aerosolized bacteriophage MS2 in comparison to no intervention. Both UV-C technologies could be useful adjunctive measures to reduce the risk for respiratory virus transmission.

## Introduction

Ultraviolet-C (UV-C) light technologies may be useful as an adjunctive measure to reduce transmission of airborne pathogens in areas with suboptimal ventilation and where aerosol-generating procedures are performed.^1^ Upper-room UV-C technologies have been used since the 1980s for control of tuberculosis,^2–4^ and have been proposed as a means to reduce transmission of measles.^5^ These devices are mounted on walls or ceilings to create a zone of disinfection above room occupants. During the coronavirus disease 2019 (COVID-19) pandemic, the Centers for Disease Control and Prevention (CDC) recommended consideration of upper room UV-C technologies in high-risk areas, including occupied areas with suboptimal ventilation.^1,6^ Several commercial upper room UV-C products are available, but relatively little information is available on their efficacy against aerosolized viruses. Moreover, there is a need to assess the potential added benefit of upper room UV-C in settings with adequate ventilation (ie, 5 or more air changes per hour)^6^ and to compare upper room 254-nm wavelength UV-C to 222-nm far UV-C technologies also proposed as an adjunct to reduce the risk for transmission of respiratory viruses.^7^

## Methods

### Test devices

The upper room UV-C device was a MED-418 (Lumalier, Inc., Memphis, TN) with four 18W UV-C lamps providing 360° delivery of 254-nm wavelength UV-C in the upper room zone. The device is intended to be used in rooms up to 40.9 m^2^. The device was suspended in the middle of the room at a height of 2.7 m.

Two far UV-C technologies were tested. The Pathogen Reduction System (Mynatek, Inc., Oakland, CA) (Device 1) uses 3 krypton-chloride excimer lamps emitting a primary wavelength of 222 nm.^8^ The 150-watt GermBuster Esconce (Sterilray, Inc., Somersworth, NH) far UV-C technology (Device 2) uses a 33 cm krypton-chloride excimer lamp that emits 222 nm light.^9^ For both far UV-C technologies, 2 devices were positioned at a height of 2.7 m on opposite sides of the room.

For comparison with the UV-C technologies, a portable high efficiency particulate air (HEPA) cleaner (Germ Guardian 5-in-1 28” Pet Pure Air Purifier with HEPA, UVC & Digital, Guardian Technologies, Euclid, OH) that processes 11.3 m^3^/min of air was tested. The device is intended to be used in rooms up to 117.6 m^2^.

### Reduction in aerosolized bacteriophage MS2

The efficacy of the technologies in reducing aerosolized bacteriophage MS2 was tested in a 48.9 m^3^ room (6.1 × 2.9 × 2.8 m) with 3 m ceiling height with the door closed. The ventilation system provided positive pressure with 6 air changes per hour. The room contained medical equipment but no large devices that would obstruct airflow. For each simulation, an Aerogen Solo (Aerogen) nebulizer was used to release 2 mL of droplets containing 10^10^ plaque-forming units (PFU) of bacteriophage MS2 over 3 minutes in the center of the room.^8^ Air samples were collected 2 m from the aerosol release site over 2 minutes at baseline and after operating the technologies for 2, 5, 15, 30, and 60 minutes using a NIOSH two stage bio-aerosol sampler (Tisch Environmental). For the upper room UV-C device, additional testing was conducted with a fan operating to increase air mixing. Quantitative cultures for bacteriophage MS2 were processed as previously described.^8^ Log_10_ reductions were calculated in comparison to control experiments in which no technology was operated. All experiments were repeated in triplicate with ≥24 hours between each experiment.

Because the far UV-C technologies demonstrated similar, substantial reductions in bacteriophage MS2 after 2 minutes of exposure, an additional evaluation was conducted with one of the far UV-C devices to assess reductions versus controls with shorter exposure times of 0.5 and 1 min. The experiments were repeated twice.

### Data analysis

A linear mixed-effects model was fitted with fixed effects for time, device, and their interaction, and a random intercept to account for each trial. This model yielded estimated marginal means for each device that were used to calculate differences between devices overall and after 60 minutes. Dunnett’s adjustment was applied to control for the familywise error rate. Data was analyzed using R version 4.2.2 software (The R Foundation for Statistical Computing, Vienna, Austria).

## Results

**Figure 1.**
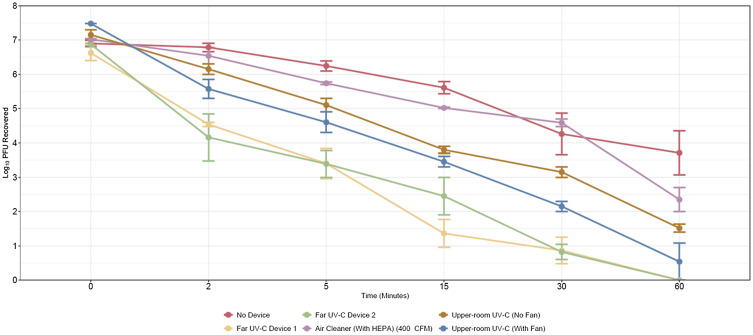
Efficacy of the upper room ultraviolet-C (UV-C), far UV-C, and a portable air cleaner in reducing aerosolized bacteriophage MS2. The upper room UV-C technology was operated with and without a room fan to increase air mixing in the room. PFU, plaque-forming units; HEPA, high efficiency particulate air cleaner; CFM, cubic feet per meter. Error bars show standard error.

As shown in Figure 1, the concentration of bacteriophage MS2 recovered from air was similar for all groups at baseline. With no technology (control), the average concentration of bacteriophage MS2 decreased gradually by 3.1-log_10_ PFU over 60 minutes. In comparison to the control, the upper room UV-C and far UV-C technologies significantly reduced recovery of bacteriophage MS2 overall (*P* ≤ .04) and after 60 minutes (mean reductions, 5.6 – 6.9 and 6.6 – 6.8 log_10_, respectively; *P* < .001 vs the control); there were no significant differences in the reductions achieved by the upper room and far UV-C technologies at 60 minutes (*P* > .22). The air cleaner significantly reduced recovery at 60 minutes (mean reduction, 4.6 log_10_; *P* = .04 vs the control) but not overall (*P* = .28). Operation of a room fan in conjunction with the upper room UV-C technology did not significantly reduce recovery of bacteriophage MS2 versus with no fan operating after 60 minutes (mean reductions, 6.9 vs 5.6 log_10_; *P* = .85). The supplemental material provides the data and statistical analyses.

**Figure 2.**
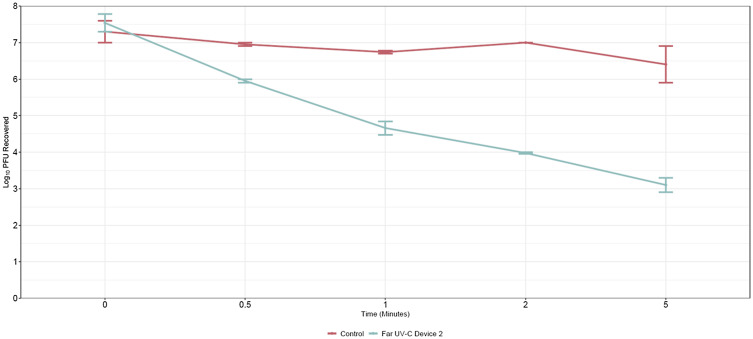
Reductions in bacteriophage MS2 achieved by a far ultraviolet-C (UV-C) technology after 0.5, 1, and 2 minutes of exposure. Error bars show standard error.

Figure 2 shows the reductions in bacteriophage MS2 achieved by far UV-C Device 2 after .5, 1, 2 and 5 minutes of exposure. The technology significantly reduced bacteriophage MS2 (*P* < .01) at each time point in comparison to controls.

## Discussion

In a room with 6 air changes per hour, recovery of aerosolized bacteriophage MS2 was reduced by ∼ 3 log_10_ over 1 hour with no added air cleaning technologies. An upper room UV-C light technology and 2 far UV-C technologies were similarly effective in further reducing aerosolized bacteriophage MS2 (5.6 – 6.9 log_10_ reduction). A portable HEPA air cleaner reduced recovery of bacteriophage MS2 in comparison to controls after 60 minutes but was less effective than the UV-C technologies. These findings suggest that upper room UV-C and far UV-C technologies may have similar efficacy as an adjunctive measure to reduce the risk for transmission of respiratory viruses in high-risk settings, including in areas with adequate ventilation.

The UV-C technologies provided a rapid reduction in bacteriophage MS2 recovery, whereas the ventilation system alone resulted in a gradual reduction (ie, 30 mins to achieve a > 2 log_10_ reduction). For 1 far UV-C technology, we demonstrated that significant reductions in bacteriophage MS2 were achieved in 30 s and 1 minute. Additional studies are needed to determine if such rapid reductions might reduce the risk for transmission of viral particles during higher-risk exposures.

Our study has some limitations. Only 1 upper room UV-C technology and 1 portable air cleaner were studied. Testing was conducted in a relatively small room with ∼ 6 air changes per hour under controlled conditions that may not account for factors impacting efficacy of the technology under real-world conditions. We did not assess the efficacy of the technologies against viral particles being dispersed repeatedly which might more closely replicate shedding by an infected person. We used a bacteriophage rather than a human pathogen. Our results may underestimate the efficacy of the UV-C technologies for reduction of enveloped viruses because bacteriophage MS2 is a non-enveloped virus that is relatively resistant to killing by UV-C.^10^ Finally, future studies are needed to determine if use of the upper room and far UV-C technologies will impact respiratory virus transmission in real-world settings.

## Supporting information

10.1017/ash.2025.10107.sm001 Memic et al. supplementary material Memic et al. supplementary material

## Supplementary material

The supplementary material for this article can be found at https://doi.org/10.1017/ash.2025.10107

## References

[1] Centers for Disease Control and Prevention. About germicidal ultraviolet (GUV). 2025. https://www.cdc.gov/niosh/ventilation/germicidal-ultraviolet/index.html?CDC_AA_refVal=https%3A%2F%2Fwww.ced.gov%2Fcoronanvirus%2F2019-ncov%2Fcommunity%2Fventilation%2FUVGI.html. Accessed May 1, 2025.

[2] Reed NG. The history of ultraviolet germicidal irradiation for air disinfection. Public Health Rep 2010;125:15–27. 10.1177/003335491012500105 PMC2789813 20402193

[3] National Center for Occupational Safety and Health. Environmental control for tuberculosis: Basic upper-room ultraviolet germicidal irradiation guidelines for healthcare. 2009. Available at: https://www.cdc.gov/niosh/docs/2009-105/default.html., Accessed May 1, 2025.

[4] Mphaphlele M , Dharmadhikari AS , Jensen PA , et al. Institutional tuberculosis transmission. Controlled trial of upper room ultraviolet air disinfection: A basis for new dosing guidelines. Am J Respir Crit Care Med 2015;192:477–84. 25928547 10.1164/rccm.201501-0060OC PMC4595666

[5] Nardell E , Nathavitharana R. Air disinfection in measles transmission hotspots. Lancet 2019;394:1009–1010. 31495498 10.1016/S0140-6736(19)31889-6

[6] Centers for Disease Control and Prevention. Ventilation in buildings, 2025. https://archive.cdc.gov/www_cdc_gov/coronavirus/2019-ncov/community/ventilation.html. Accessed April 19, 2025.

[7] Blatchley ER III , Brenner DJ , Claus H , et al. Far UV-C radiation: an emerging tool for pandemic control. Crit Rev Env Sci Technol 2022;53:733–753. doi: 10.1080/10643389.2022.2084315

[8] Memic S , Osborne AO , Cadnum JL , Donskey CJ. Efficacy of a far-ultraviolet-C light technology for continuous decontamination of air and surfaces. Infect Control Hosp Epidemiol 2024;45:132–134. 37529841 10.1017/ice.2023.159

[9] Osborne AO , Memic S , Cadnum JL , Donskey CJ. Evaluation of a wall-mounted far ultraviolet-C light device used for continuous air and surface decontamination in a dental office during routine patient care. Infect Control Hosp Epidemiol 2024;45:1250–1252. doi: 10.1017/ice.2024.109 39228210

[10] Cadnum JL , Li DF , Redmond SN , John AR , Pearlmutter B , Donskey CJ. Effectiveness of ultraviolet-C light and a high-level disinfection cabinet for decontamination of N95 respirators. Pathog Immun 2020;5:52–67. 32363254 10.20411/pai.v5i1.372 PMC7192214

