# Supplementary material for: Efficacy of an upper room ultraviolet-C (UV-C) technology versus far UV-C light technologies in reducing aerosolized bacteriophage MS2
Source: Antimicrob Steward Healthc Epidemiol. 2025 Sep 15;5(1):e221. doi: 10.1017/ash.2025.10107 (PMC12451806; doi:10.1017/ash.2025.10107)
Supplement: Memic et al. supplementary material [file S2732494X25101071sup001.docx]

**Supplementary material**

**Title: Efficacy of an upper room ultraviolet-C (UV-C) technology versus far UV-C light technologies in reducing aerosolized baceriophage MS2** (**ASHE-D-25-00173)**

**
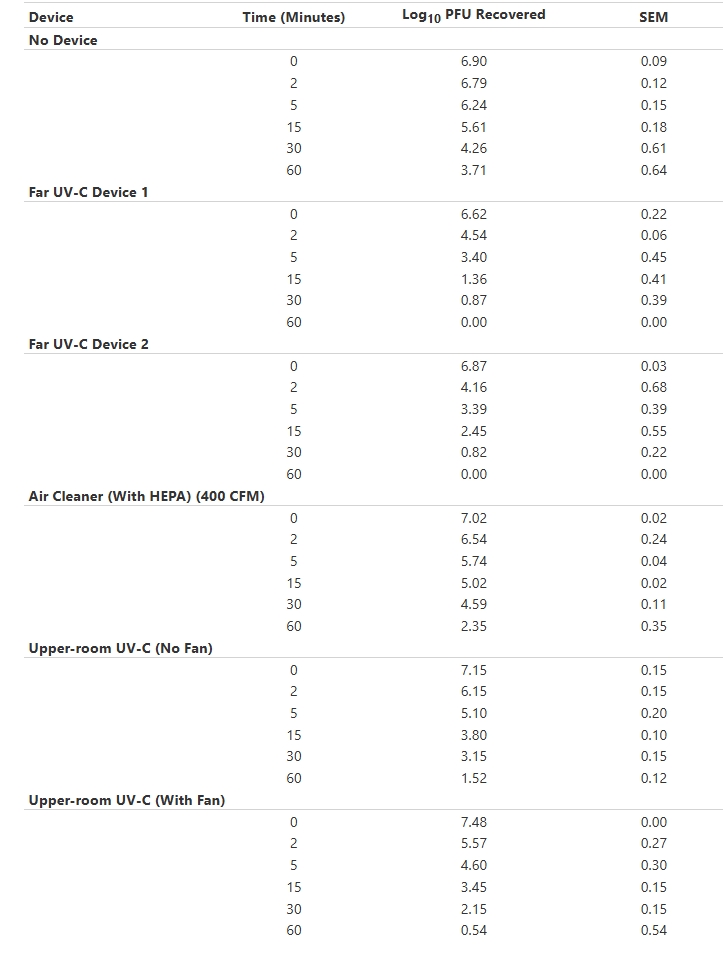
**

**Overall Effect of Devices in Reducing Aerosolized Bacteriophage MS2**


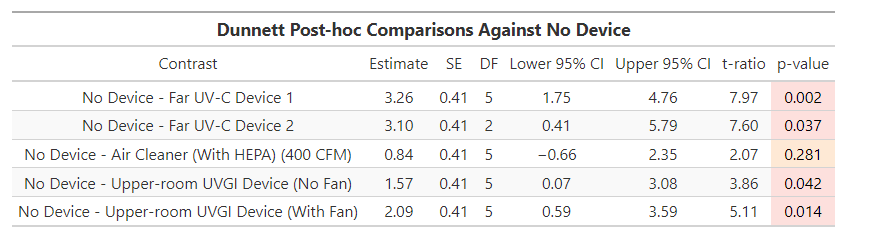


The first figure presents the Dunnett post-hoc estimated contrasts in MS2 concentration between each device and no device (control). Aggregated across all time points. These contrasts reflect the overall mean difference in bacteriophage concentration between the control group and the devices. Far UV-C device 1 (Mean Difference = 3.26; 95 CI [1.75, 4.76]; *P* = 0.002) and far UV-C device 2 (MD = 3.10; 95 CI [0.41, 5.79]; *P* = 0.037) produced significantly large reductions in MS2 bacteriophage compared to controls. The upper-room UVGI device has also had significant impact on MS2 bacteriophage concentrations within the room. When used without a fan the UVGI device reduced bacteriophage concentrations by 1.57 log10 PFU (95% CI [0.13, 3.02], *P* = 0.035), however, when a fan was added to the room to stimulate airflow, the device had increased efficacy of reducing airborne particles (MD = 2.09; 95% CI [0.64, 3.54]; *P* = 0.035). The air cleaner with a HEPA filter, was the only device that had a positive but non-significant overall difference in reducing MS2 concentrations (MD = 0.84; 95% CI [-0.61, 2.29]; *P* = 0.290) across all of the timepoints.

**Pairwise Comparisons for Each Device at 60 Minutes**

**
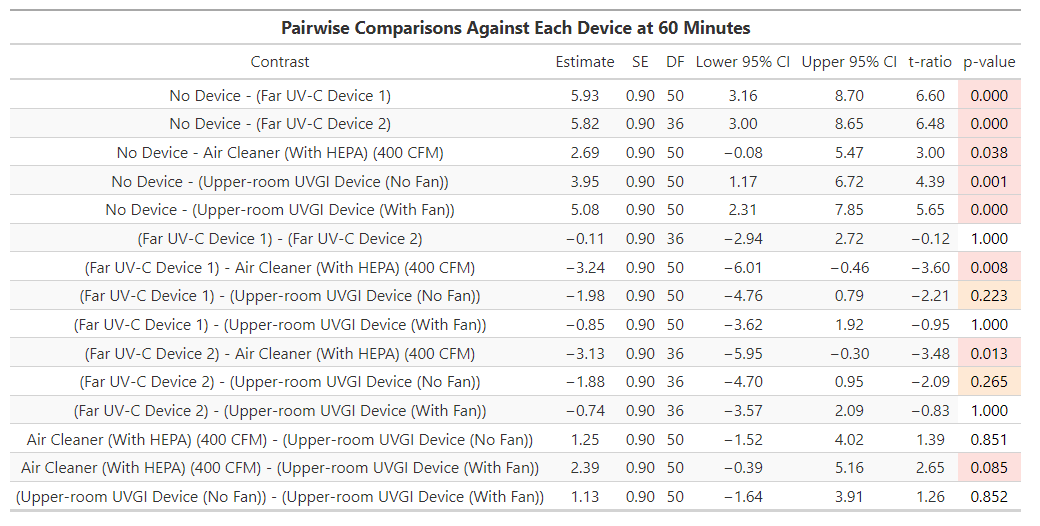
**

Additionally, pairwise contrasts we done between all devices at 60 minutes. At 60 minutes, all of the devices (all *P* < 0.001) had significant reductions in airborne bacteriophage concentrations All of the far UV-C devices (Device 1 and 2) had similar reductions to one another and were not significantly different from each other (*P* > 0.99). Far UV-C device 1 resulted in significantly lower concentrations of airborne MS2 compared to the air cleaner (MD = -3.24; 95% CI [-0.37, -3.59]; *P* = 0.011). A similar trend was found when comparing as did Far UV-C device 2 (MD = -3.13; 95% CI [-5.95, -0.30]; *P* = 0.013) to the control. Notably, when comparing the upper-room UVGI without a fan to the upper-room UVGI device with a fan, there was a decreasing but non-significant difference at 60 minutes (MD = 1.13; 95% CI [-1.64, 3.91], *P* > 0.85). These finding suggests that additional air flow in an already ventilated space may provide minimal benefit to upper-room UVGI devices.

**Trial with Far UV-C Device 2 with Short Exposure Times**


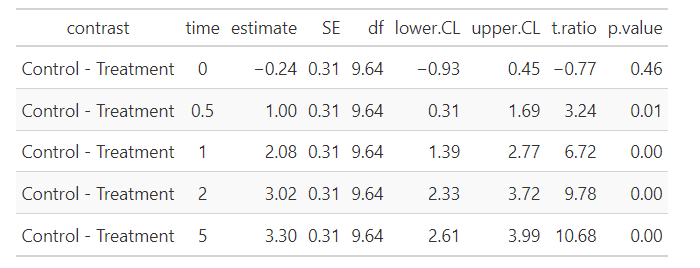


Post-hoc pairwise contrasts for a linear mixed model compared the difference between the control and treatment devices at the 0, 0.5, 1, 2, and 5 minutes. At baseline there was no significant difference in airborne MS2 immediately after release (MD = -0.24; 95% CI [-0.93, 0.45]; *P* = 0.46), indicating a similar starting concentration between both trials. After treatment with far UV-C, the device achieved a significant 1.0 log_10_ PFU reduction in MS2 (95% CI [0.31, 1.69; *P* = 0.01) compared to the control. After one minute, the device achieved a 2.08 log_10_ PFU reduction (95% CI [1.39, 2.77]; *P* < 0.001). A further reduction was observed at two (MD = 3.02; 95% CI [2.33, 3.72]; *P* < 0.001) and three minutes (MD = 3.30; 95% CI [2.61, 3.99]; *P* < 0.001). These result demonstrate that the far UV-C devices can produce rapid and substantial inactivation of airborne MS2, achieving a >1 log PFU reduction within 30 seconds of treatment time, and exceeding a 3 log_10_ PFU reduction after 2 minutes of treatment time.
